# Decrease in Sleep Duration and Poor Sleep Quality over Time Is Associated with an Increased Risk of Incident Non-Alcoholic Fatty Liver Disease

**DOI:** 10.3390/jpm12010092

**Published:** 2022-01-11

**Authors:** Yoo Jin Um, Yoosoo Chang, Hyun-Suk Jung, In Young Cho, Jun Ho Shin, Hocheol Shin, Sarah H. Wild, Christopher D Byrne, Seungho Ryu

**Affiliations:** 1 Total Healthcare Center, Kangbuk Samsung Hospital, Sungkyunkwan University School of Medicine, Seoul 04514, Korea; yoojin.um@samsung.com (Y.J.U.); hs1601.jung@samsung.com (H.-S.J.); inyoungs.cho@samsung.com (I.Y.C.); junho0521.shin@samsung.com (J.H.S.); hcfm.shin@samsung.com (H.S.); 2 Center for Cohort Studies, Total Healthcare Center, Kangbuk Samsung Hospital, Sungkyunkwan University School of Medicine, Seoul 04514, Korea; 3 Department of Occupational and Environmental Medicine, Kangbuk Samsung Hospital, Sungkyunkwan University School of Medicine, Seoul 03181, Korea; 4 Department of Clinical Research Design & Evaluation, SAIHST, Sungkyunkwan University, Seoul 06355, Korea; 5 Department of Family Medicine, Kangbuk Samsung Hospital, Sungkyunkwan University School of Medicine, Seoul 03181, Korea; 6 Department of Surgery, Kangbuk Samsung Hospital, Sungkyunkwan University School of Medicine, Seoul 03181, Korea; 7 Usher Institute, University of Edinburgh, Edinburgh EH8 9AG, UK; sarah.wild@ed.ac.uk; 8 Nutrition and Metabolism, Faculty of Medicine, University of Southampton, Southampton SO16 6YD, UK; C.D.Byrne@soton.ac.uk; 9 National Institute for Health Research Southampton Biomedical Research Centre, University Hospital Southampton, Southampton SO16 6YD, UK

**Keywords:** hepatic steatosis, hepatic fibrosis, change in sleep duration, sleep quality, Pittsburgh sleep quality index, fibrosis-4 score

## Abstract

The impact of changes in sleep duration and sleep quality over time on the risk of non-alcoholic fatty liver disease (NAFLD) is not known. We investigated whether changes in sleep duration and in sleep quality between baseline and follow-up are associated with the risk of developing incident NAFLD. The cohort study included 86,530 Korean adults without NAFLD and with a low fibrosis score at baseline. The median follow-up was 3.6 years. Sleep duration and quality were assessed using the Pittsburgh Sleep Quality Index. Hepatic steatosis (HS) and liver fibrosis were assessed using ultrasonography and the fibrosis-4 index (FIB-4). Cox proportional hazard models were used to determine hazard ratios (HRs) and 95% confidence intervals (Cis). A total of 12,127 subjects with incident HS and 559 with incident HS plus intermediate/high FIB-4 was identified. Comparing the decrease in sleep duration of >1 h, with stable sleep duration, the multivariate-adjusted HR (95% CIs) for incident HS was 1.24 (1.15–1.35). The corresponding HRs for incident HS plus intermediate/high FIB-4 was 1.58 (1.10–2.29). Comparing persistently poor sleep quality with persistently good sleep quality, the multivariate-adjusted HR for incident HS was 1.13 (95% CI, 1.05–1.20). A decrease in sleep duration or poor sleep quality over time was associated with an increased risk of incident NAFLD, underscoring an important potential role for good sleep in preventing NAFLD risk.

## 1. Introduction

Non-alcoholic fatty liver disease (NAFLD), the most common cause of chronic liver disease, is a multisystem disease associated with a risk of hepatic and non-hepatic complications including cardio-metabolic disorders [1,2,3,4]. A lifestyle modification, such as weight loss, is considered the first-line treatment as there is no approved drug for NAFLD treatment [5,6]. It is important to evaluate all modifiable lifestyle factors, such as sleep duration and quality, to establish a preventive strategy to reduce NAFLD risk.

We spend approximately one-third of our lifetime asleep and good quality sleep is crucial for our cardiovascular health and the regulation of endocrine and immune systems [7,8]. The National Sleep Foundation has reported the importance of sleeping more than 7 h per day for adults to maintain ideal health [9]. However, in recent decades, the prevalence of short sleep duration (defined as <6 h) has been reported to be over 20% [10]. Epidemiological studies suggest that short sleep duration is associated with obesity, metabolic syndrome, and cardiovascular diseases [11,12]. These conditions are also commonly seen in patients with NAFLD [13], although two meta-analyses investigating the relationship between sleep duration and NAFLD showed conflicting results [14,15].

Sleep duration is affected by various factors and can change over time [16]. According to one meta-analysis, the total amount of sleep decreased dramatically with age in adults and the change gradually disappeared in the elderly [17]. Further, the intra-individual variability of sleep was greater in younger adults than in older adults [18]. In modern society, owing to the choice of lifestyle, family demand, or work-related factors, the prevalence of intra-individual difference in sleep duration is increasing [19,20]. Therefore, the influence of sleep duration on health conditions cannot be fully determined without considering changes in sleep duration over time [21]. Several cohort studies have reported an association between changes in sleep duration and adverse health outcomes, including metabolic syndrome [21], type 2 diabetes [22], and mortality [23]. However, to date, no study has investigated the impact of changes in sleep duration and in sleep quality over time on the risk of NAFLD. Our previous study demonstrated an association between sleep duration and sleep quality as risk factors for NAFLD, but in our previous work, we did not evaluate the role of change in sleep as a risk factor for NAFLD [24]

We aimed to evaluate the relationship between changes in sleep duration and in sleep quality and the subsequent development of NAFLD, both with and without intermediate/high probability of liver fibrosis, whilst accounting for time-dependent measures including changes in sleep duration, changes in sleep quality, and potential confounders during the follow-up period.

## 2. Methods

### 2.1. Study Population

**Figure 1:**
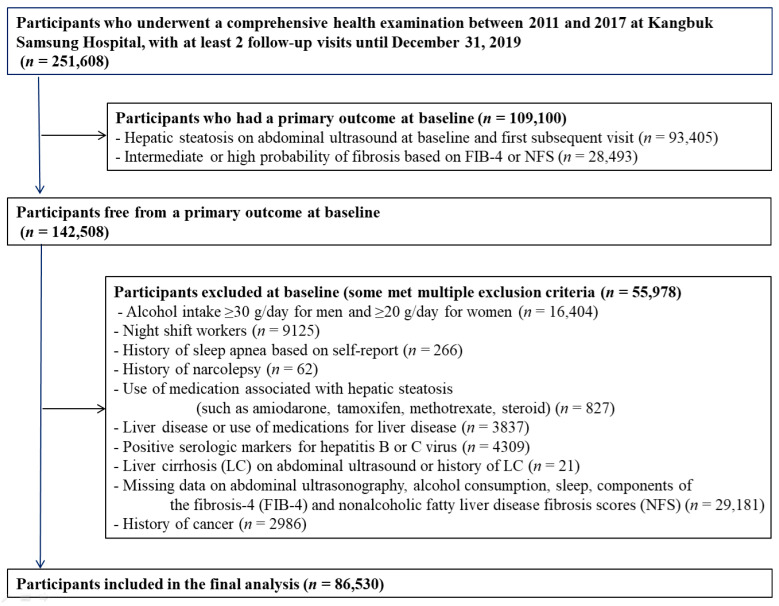
Flowchart of study participants.

This cohort study is a part of the Kangbuk Samsung Health Study, a cohort study of Korean adults who participated in a health examination annually or biennially at Kangbuk Samsung Hospital Total Healthcare Centers in Seoul and Suwon, South Korea [25,26]. The present study population was restricted to individuals who underwent baseline and subsequent health screening examinations with information on sleep duration and sleep quality from March 2011 to December 2017 and had at least one follow-up visit by 31 December 2019 (N = 251,608). We excluded subjects who had either hepatic steatosis (HS) or intermediate/high fibrosis-4 (FIB-4) scores at baseline or subsequent visits (n = 109,100). Then, we excluded 55,978 subjects who met one or more of the exclusion criteria at baseline (Figure 1). The final sample included 86,530 subjects. This study was approved by the Institutional Review Board of Kangbuk Samsung Hospital (IRB no. 2021-01-024) and was conducted in accordance with the Declaration of Helsinki. The requirement for informed consent was waived owing to the use of a preexisting de-identified dataset that was routinely collected during the health screening process.

### 2.2. Data Collection

All baseline and follow-up examinations were conducted at Kangbuk Samsung Hospital Health Screening Center clinics. The data regarding patients’ demographic characteristics, behavioral factors, medical history, and medication use were collected using a standardized, self-administered questionnaire, while anthropometry, blood pressure, and serum biochemical parameters were measured by trained staff during the health examination. Depressive symptoms were assessed using the Korean version of the Center for Epidemiologic Studies Depression (CES-D) scale and were categorized as having CES-D scores < 16 or ≥16 [27,28].

Sleep duration and quality were assessed using the validated Pittsburgh Sleep Quality Index (PSQI) at baseline and during the follow-up sessions [29]. The PSQI is a validated 19-item self-administered questionnaire used to evaluate sleep quality during the previous month. The PSQI consists of seven components: subjective sleep quality, sleep latency, sleep duration, habitual sleep efficiency, sleep disturbances, use of sleeping medication, and daytime function. Each component score ranged from 0 (best) to 3 (worst sleep properties), and the PSQI score was calculated as the sum of each component score to generate an overall score. In one of the PSQI items, the subjects were asked to report the hours of actual sleep at night in a typical 24 h period over the previous month. Sleep duration was categorized into ≤5, 6, 7, 8, and ≥9 h. The changes in sleep duration were calculated for each subject as the difference in sleep duration between baseline and subsequent visit (visit 1 and visit 2) values; these changes were categorized into the following five groups: (1) decrease in sleep duration of > 1 h, (2) decrease in sleep duration of 0.1 to 1 h, (3) 0 (stable sleep duration, reference), (4) increase in sleep duration of 0.1 to 1 h, and (5) increase in sleep duration of ≥1 h. Poor sleep quality was defined as a PSQI score of ≥ 6, and good sleep quality was defined as a PSQI score of < 6. Changes in sleep quality were categorized into the following four groups: (1) persistently good sleep quality (good sleep quality at both baseline and follow-up (reference group), (2) good sleep quality at baseline but newly developed poor sleep quality at follow-up, (3) poor sleep quality at baseline but good sleep quality at follow-up, and (4) persistently poor sleep quality (poor sleep quality at both baseline and follow-up).

The diagnosis of HS was based on an abdominal ultrasound, performed by an experienced radiologist who was blind to the aim of the present study. This diagnosis was determined using standard criteria, including the presence of a diffuse increase in fine echoes in the liver parenchyma compared with those of the kidney or spleen parenchyma, deep beam attenuation, and bright vessel walls [30]. The inter-observer and intra-observer reliability values for HS diagnoses were substantial (kappa statistic of 0.74) and excellent (kappa statistic of 0.94), respectively [26].

To assess the risk of progression to more severe NAFLD, a non-invasive index of liver fibrosis, FIB-4, was used [31]. The subjects were classified into three groups, reflecting the probability of advanced fibrosis based on the FIB-4 score: low (FIB-4 <1.30), intermediate (FIB-4 1.30–2.66), and high (FIB-4 ≥ 2.67) [31].

### 2.3. Statistical Analysis

The baseline characteristics of the subjects were described according to the changes in sleep duration.

The primary endpoints were the development of (a) incident HS and (b) incident HS plus an intermediate/high probability of liver fibrosis. Incident HS and incident HS combined with an intermediate/high probability of liver fibrosis based on FIB-4 were treated as separate endpoints in each model. The event detection date was defined as the earliest date of identification of HS or HS with an intermediate or high probability of liver fibrosis based on the FIB-4 score, which was analyzed separately. The person-years were calculated as the sum of the follow-up duration from baseline to the event detection date (HS or HS with fibrosis, separately) or until the final examination (before 31 December 2019), whichever occurred first. Hazard ratios (HRs) and 95% confidence intervals (CIs) were calculated using a Cox proportional hazards model.

The risks of incident HS and incident HS combined with an intermediate/high probability of liver fibrosis were separately evaluated according to the changes in sleep duration. The models were initially adjusted for age and sex. Then, they were further adjusted for the following additional potential confounders: study center (Seoul, Suwon), year of the screening examination, season (spring, summer, fall, and winter), smoking status (never, past, current, or unknown), alcohol intake (none, <10, or ≥10 g/day, or unknown), physical activity (inactive, minimally active, health-enhancing physical activity [HEPA], or unknown), marital status, history of diabetes, history of hypertension, sleep duration at baseline, and sleep quality (for the analysis of changes in sleep duration); Model 1). Next, we sought to examine whether the relationship between sleep duration and development of the primary endpoints was mediated by body mass index (BMI; Model 2) on a priori grounds. We evaluated the mediation effect of BMI on the association between sleep duration and the risk of HS or HS plus an intermediate/high probability of liver fibrosis if the BMI met the three criteria for being a potential mediator as follows: (1) change in sleep duration was associated with BMI, (2) BMI was significantly associated with the incident endpoint when change in sleep duration was included in the model, and (3) the addition of BMI to the model attenuated the association between change in sleep duration and incident HS. We assessed the proportional hazards assumption by examining graphs of estimated log (−log(survival)) versus the log of the survival time graph and found no violation of the assumption.

Statistical analyses were performed using STATA version 16.0 (StataCorp LP, College Station, TX, USA). All reported *p*-values were two-tailed, and a *p*-value < 0.05 was considered statistically significant.

## 3. Results

Table 1 shows the baseline characteristics of the 86,530 subjects according to changes in sleep duration. At baseline, the mean (SD) age and the median change in sleep duration were 36.5 (6.0) years and 0 (interquartile range, −1 to 1) hour, respectively. Compared with subjects with stable sleep duration, those with either a decrease or increase in sleep duration between baseline and subsequent visits were more likely to be younger, have depressive symptoms, and less likely to be men and current smokers.

During the 305,833 person-year follow-up, 12,127 cases of incident HS were identified (incidence rate 39.7/103 person-years). The median follow-up duration was 3.6 years (interquartile range, 2.0–5.0). A decrease in sleep duration was associated with an increased risk of incident HS (Table 2). After adjusting for age, sex, center, year of the screening examination, season, alcohol consumption, smoking, physical activity, marital status, history of diabetes, history of hypertension, sleep duration, and sleep quality, the multivariate-adjusted HR (95% CIs) for incident HS comparing changes in sleep durations of <−1, −1 to 0.1, 0.1 to 1, and >1 h with 0 h (reference) was 1.24 (1.15–1.35), 1.12 (1.06–1.17), 1.00 (0.95–1.05), and 0.99 (0.91–1.08), respectively. After further adjusting for BMI (Model 2), the association between the decrease in sleep duration and incident HS was attenuated but remained significant. After adjusting for WC instead of BMI, this association persisted (Supplementary Table S1). Compared with persistently good sleep quality, persistently poor sleep quality was associated with an increased risk of incident HS. After adjusting for BMI, sleep duration, and other confounders, the multivariate-adjusted HR comparing persistently poor sleep quality with persistently good sleep quality was 1.13 (95% CI, 1.05–1.20). The resolution of poor sleep quality or newly developed poor quality was not associated with the risk of HS.

During the 332,785.9 person-year follow-up, 559 cases of incident HS plus an intermediate/high FIB-4 were identified (incidence rate 1.7/10^3^ person-years). After adjusting for age, sex, and other confounders, the multivariate-adjusted HR (95% CI) for incident HS plus an intermediate/high FIB-4 comparing change in sleep durations of <−1, −1 to 0.1, 0.1 to 1, and >1 h with 0 h (stable sleep duration, the reference) was 1.58 (1.10–2.29), 1.16 (0.94–1.44), 0.98 (0.77–1.23), and 0.89 (0.56–1.42), respectively. (Table 3) After further adjusting for BMI (Model 2), the association between a decrease in sleep duration of >1 h and incident HS plus intermediate/high FIB-4 remained significant. Compared with persistently good sleep quality, persistently poor sleep quality tended to be associated with an increased risk of HS plus intermediate/high FIB-4, but this did not reach significance.

## 4. Discussion

In this large-scale prospective cohort study of 86,530 patients with a median age of 36.5 years, a decrease in sleep duration over time and persistently poor sleep quality were associated with an increased risk of developing NAFLD both with and without an intermediate/a high fibrosis score. After further adjusting for BMI, the association between decreased sleep and NAFLD was attenuated but remained significant. Furthermore, compared with persistently good sleep quality, persistently poor sleep quality was significantly associated with the risk of NAFLD even after adjusting for BMI. This trend was similarly seen in the relationship between a decrease in sleep duration and incident HS plus an intermediate/high FIB-4. Persistent poor sleep quality also tended to be associated with an increased risk of HS plus intermediate/high FIB-4, but the relationship was not significant.

Currently, no cohort studies are available on the relationship between sleep changes and NAFLD risk. A cohort study of 15,753 participants in China found an association between shortening of sleep duration and the risk of metabolic syndrome [21]. Another cohort study in the UK showed an association between increased sleep duration and the risk of type 2 diabetes [22]. In the same study, the increased risk was also associated with a decrease in sleep duration over time, although this was not significant, possibly due to the insufficient number of participants examined for the change in sleep duration. In addition, sleep quality, which would have been helpful in determining whether the long sleep duration was compensatory, was not analyzed. Finally, another cohort study with 9781 participants showed a U-shaped relationship between sleep duration change and mortality, indicating both a decrease and increase in sleep duration as a predictor of increased mortality [23].

The present study is the first to show an association between the decrease in sleep duration and the increased risk of incident NAFLD, which extends and is in agreement with results from previous studies [13]. Ref. [21]. Furthermore, whilst none of these previous studies considered change in sleep quality, our study also incorporated change in sleep quality over time as a key exposure, extending the work of others in this field.

There are some plausible mechanisms linking the decrease in sleep and NAFLD. Hypothalamic–pituitary–adrenal (HPA) axis and autonomic nervous system activities are important in the regulation of the immune system and cardiometabolic function [32]. Cortisol, inflammatory cytokines, and norepinephrine, which are derivatives of these systems, are associated with the variation in sleep [33]. The dysregulation of HPA axis caused by changes in sleep increases the risk of chronic diseases [34]. Further, the individual behavioral factors also need to be considered. After several days of sleep deprivation due to workload or school load, there is a tendency for individuals to seek sleep compensation by sleeping more on weekends or drinking caffeine [16,17]. These behaviors may impair sleep the following night, further provoking an instability and variation in sleep [33]. Consequential poor sleep quality may induce an elevated risk of cardiovascular disease, obesity, and other comorbidities [35].

Considering the deprivation of sleep itself, a decline in sleep induces appetite through the increase in ghrelin and decrease in leptin levels [36]. This eventually triggers weight gain and obesity, which is a risk factor for NAFLD [37]. Further, a deprivation in sleep can also cause impaired insulin sensitivity [38], and insulin resistance is a key factor in the pathogenesis of NAFLD. Moreover, proinflammatory activity, such as an increase in IL-6 or TNF-α, can be aggravated by the decrease in sleep. [38,39] This relationship is significant because inflammation, induced by inflammatory activity, is another mechanism of NAFLD. Additionally, the suppression of melatonin, known as a strong antioxidant, may provoke chronic inflammation, increasing the risk of liver disease and other chronic diseases, including cardiovascular diseases [33,40]

In our large-scale cohort study, we evaluated the effect of changes in sleep duration and quality on NAFLD both with and without fibrosis, which is a major strength of our study. In addition, the participants of our study comprised a relatively young population, which decreases potential bias from possible comorbidities. Furthermore, to the best of our knowledge, our study is the first to analyze the relationship of the change in sleep duration and quality with NAFLD. As NAFLD is one of the most frequently seen chronic liver diseases and the prevalence of short sleep duration is approximately 20% [10], the results of our study suggest the importance of maintaining adequate sleep duration and good sleep quality in public health.

## 5. Limitations

There are some limitations to our study. First, sleep duration was assessed using a self-administered questionnaire. However, self-reported sleep evaluation is commonly used in many studies and self-assessments are known to be moderately correlated with actigraphy or objectively measured sleep duration [41,42]. Further, we used the widely validated PSQI to analyze sleep quality [29]. Second, a histologic diagnosis of the liver was not made. Histologic assessments are accurate in evaluating the severity of steatosis, but an ultrasonography is commonly used in many cohort studies and is also an acceptable modality in the diagnosis of fatty liver [43]. Recently, newer non-invasive methods for the assessment of both hepatic steatosis and fibrosis have been developed and validated. One such promising technique that is becoming available in clinical practice is multiparametric ultrasound. Multiparametric ultrasound utilizes ultrasound, shear wave elastography and contrast-enhanced ultrasound measurements and this methodology may be particularly useful in large cohort studies as it provides a non-invasive assessment of both liver steatosis and fibrosis in NAFLD [44,45]. Third, the possible reason for the change in sleep length was not evaluated. The decrease in duration over time may be caused by either intentional or unintentional factors, or both. These include workload, stress, sleep apnea, comorbidities, or unknown underlying diseases, and further studies considering these two factors are needed. Finally, the large proportion of young patients in our study may limit the generalizability to other age or ethnic groups.

## 6. Conclusions

Our results show that a decrease in sleep duration and poor sleep quality over time is associated with an increased risk of incident NAFLD. Further studies evaluating the interventional effects of modifying sleep duration are required.

### 6.1. Key Points

#### 6.1.1. Question

Do changes in sleep duration and sleep quality independently affect the risk of non-alcoholic fatty liver disease (NAFLD)NAFLD?

#### 6.1.2. Findings

In this large-scale prospective cohort study of 86,530 patients, stable sleepers had the lowest risk of incident hepatic steatosis (HS) and HS plus an intermediate/high FIB-4 score. A decrease in sleep duration over time was significantly associated with an increased risk of both incident HS and HS plus an intermediate/high FIB-4 score. Compared with persistently good sleep quality, persistently poor sleep quality was associated with an increased risk of HS and HS plus an intermediate/high FIB-4.

#### 6.1.3. Meaning

The maintenance of adequate sleep duration and good sleep quality should be considered as a preventive strategy for reducing NAFLD risk and its consequences. Physicians should be observant of changes in sleep duration and sleep quality, which might be a good timing to help identify individuals at high risk of subsequent NAFLD.

## Figures and Tables

**Table 1:** Baseline characteristics of study participants by sleep duration.

Characteristics	Overall	Sleep Duration Change Category (Hours)				
		<−1	−1	0	1	>1
Number	86,530	5991	19,112	37,981	18,091	5355
Age (years) ^a^	36.5 (6.0)	35.5 (5.5)	36.6 (5.9)	37.0 (6.1)	36.1 (5.9)	35.1 (5.7)
Men (%)	38.7	23.4	37.7	44.0	38.1	23.4
Obesity (%)	9.6	8.6	10.0	10.0	9.0	8.2
Current smoker (%)	13.3	9.4	13.4	14.7	13.0	8.6
Alcohol intake (%) ^c^	23.5	18.2	23.0	24.9	23.7	19.1
HEPA (%)	13.8	13.6	14.3	13.9	13.4	12.6
High education (%) ^d^	88.9	86.5	88.3	89.4	89.5	88.0
Married (%)	80.5	85.8	82.0	79.8	78.5	81.0
Depression (%)	11.2	13.7	10.4	10.1	11.8	17.6
Hypertension	3.8	2.7	3.7	4.2	3.7	2.5
Diabetes	0.6	0.5	0.6	0.6	0.5	0.4
History of CVD	0.7	0.4	0.7	0.8	0.6	0.5
BMI (kg/m^2^)	21.6 (2.5)	21.4 (2.5)	21.6 (2.6)	21.7 (2.5)	21.6 (2.5)	21.3 (2.5)
Systolic BP (mmHg) ^a^	104.4 (11.6)	102.5 (11.3)	104.5 (11.7)	105.1 (11.7)	104.2 (11.5)	101.9 (10.8)
Diastolic BP (mmHg) ^a^	66.7 (8.8)	65.5 (8.6)	66.8 (8.8)	67.2 (8.9)	66.5 (8.7)	65.2 (8.2)
Glucose (mg/dL) ^a^	91.2 (8.8)	90.5 (8.1)	91.4 (8.9)	91.5 (8.8)	91.0 (8.9)	90.0 (8.4)
Total cholesterol (mg/dl) ^a^	186.4 (31.1)	184.9 (31.4)	186.5 (31.4)	187.0 (31.1)	186.2 (30.9)	183.7 (30.4)
LDL-C (mg/dL) ^a^	111.8 (28.8)	109.6 (28.2)	111.8 (28.9)	112.8 (29.2)	111.7 (28.7)	108.3 (27.9)
HDL-C (mg/dL) ^a^	63.2 (14.6)	64.0 (14.6)	63.1 (14.5)	62.8 (14.5)	63.5 (14.6)	64.6 (14.6)
Triglycerides (mg/dl) ^b^	73 (56–100)	72 (55–97)	73 (56–100)	75 (57–102)	73 (56–98)	69 (54–93)
ALT (U/L) ^b^	14 (11–19)	13 (10–18)	14 (11–19)	15 (11–20)	14 (11–19)	13 (11–18)
GGT (U/L) ^b^	15 (11–22)	13 (10–19)	15 (11–22)	16 (11–23)	15 (11–22)	13 (10–19)
HOMA-IR ^b^	1.00 (0.68–1.41)	1.01 (0.68–1.45)	1.01 (0.68–1.42)	0.99 (0.67–1.41)	0.99 (0.68–1.42)	0.98 (0.65–1.40)
hsCRP (mg/L) ^b^	0.3 (0.2–0.6)	0.3 (0.2–0.6)	0.3 (0.2–0.6)	0.3 (0.2–0.6)	0.3 (0.2–0.6)	0.3 (0.2–0.6)
Total energy intake ^b^^,e^	1487 (1137–1863)	1469 (1109–1839)	1493 (1144–1868)	1500 (1155–1867)	1469 (1114–1851)	1463 (1104–1898)
Poor sleep quality	19.3	18.8	16.0	16.0	23.5	39.9

Data are expressed as ^a^ mean (standard deviation), ^b^ median (interquartile range), or percentage. Abbreviations: ALT, alanine aminotransferase; BMI, body mass index; BP, blood pressure; CVD, cardiovascular disease; HDL-C, high-density lipoprotein cholesterol; HEPA, health-enhancing physical activity; hsCRP, high-sensitivity C-reactive protein; HOMA-IR, homeostasis model assessment of insulin resistance. ^c^ ≥10 g of ethanol per day; ^d^ ≥college graduate; ^e^ among 63,403 participants with plausible estimated energy intake levels (within three standard deviations from the log-transformed mean energy intake).

**Table 2:** Hazard ratios (95% CIs) of incident hepatic steatosis per sleep duration change and subjective sleep quality change.

	Person-Years (PY)	Incident Cases	Incidence Rate (/1000 PY)	Age and Sex-Adjusted HR (95% CI)	Multivariable-Adjusted HR ^a^ (95% CI)	
					Model 1	Model 2
**Sleep Duration Change Category**						
<−1 h	21,758.4	760	34.9	1.13 (1.05–1.22)	1.24 (1.15–1.35)	1.14 (1.06–1.24)
−1 h	69,109.2	2788	40.3	1.07 (1.02–1.12)	1.12 (1.06–1.17)	1.07 (1.02–1.12)
0 h	134,225.6	5519	41.1	1.00 (reference)	1.00 (reference)	1.00 (reference)
1 h	62,399.4	2453	39.3	1.05 (1.00–1.10)	1.00 (0.95–1.05)	1.02 (0.97–1.07)
>1 h	18,340.3	607	33.1	1.09 (1.00–1.19)	0.99 (0.91–1.08)	1.03 (0.94–1.12)
P for trend				0.195	< 0.001	0.015
P for quadratic term				0.003	< 0.001	0.018
**Sleep quality change category**						
Persistent good quality	218,435.7	9076	41.5	1.00 (reference)	1.00 (reference)	1.00 (reference)
Developed poor quality	29,805.7	1038	34.8	1.02 (0.95–1.09)	1.00 (0.94–1.07)	1.02 (0.95–1.08)
Resolved poor quality	29,182.2	987	33.8	1.00 (0.93–1.06)	0.96 (0.90–1.02)	1.00 (0.93–1.07)
Persistent poor quality	28,409.4	1026	36.1	1.10 (1.03–1.17)	1.05 (0.98–1.12)	1.13 (1.05–1.20)

Estimated from Cox proportional hazards models. The multivariate model was adjusted for age, sex, center, year of screening examination, alcohol consumption, smoking, physical activity, marital status, season, history of diabetes, history of hypertension, sleep quality (only for sleep duration change category), and sleep duration at baseline; model 2: model 1 plus adjustment for BMI. Abbreviations: BMI, body mass index; CI, confidence interval; HR, hazard ratio.

**Table 3:** Hazard ratios (95% CIs) of incident hepatic steatosis plus intermediate/high probability of advanced fibrosis based on FIB-4 with respect to sleep duration change and subjective sleep quality change.

.	Person-Years (PY)	Incident Cases	Incidence Rate (/1000 PY)	Age and Sex-Adjusted HR (95% CI)	Multivariable-Adjusted HR ^a^ (95% CI)	
					Model 1	Model 2
**Sleep Duration Change Category**						
<−1 h	23,439.9	36	1.54	1.37 (0.96–1.94)	1.58 (1.10–2.29)	1.45 (1.004–2.10)
−1 h	75,475.2	130	1.72	1.09 (0.89–1.35)	1.16 (0.94–1.44)	1.11 (0.90–1.38)
0 h	146,570.2	268	1.83	1.00 (reference)	1.00 (reference)	1.00 (reference)
1 h	67,695.1	104	1.54	1.03 (0.82–1.29)	0.98 (0.77–1.23)	0.99 (0.79–1.25)
>1 h	19,605.6	21	1.07	1.00 (0.64–1.56)	0.89 (0.56–1.42)	0.93 (0.58–1.49)
P for trend				0.197	0.028	0.104
P for quadratic term				0.509	0.381	0.543
**Sleep quality change category**						
Persistent good quality	238,838	429	1.8	1.00 (reference)	1.00 (reference)	1.00 (reference)
Developed poor quality	32,044.1	46	1.4	1.13 (0.83–1.53)	1.11 (0.82–1.51)	1.13 (0.83–1.53)
Resolved poor quality	31,347	39	1.2	0.97 (0.70–1.35)	0.92 (0.66–1.29)	0.96 (0.69–1.34)
Persistent poor quality	30,556.8	45	1.5	1.17 (0.86–1.59)	1.09 (0.79–1.49)	1.18 (0.86–1.62)

Estimated from Cox proportional hazards models. The multivariate model was adjusted for age, sex, center, year of screening examination, alcohol consumption, smoking, physical activity, marital status, season, history of diabetes, history of hypertension, sleep quality (only for sleep duration change category), and sleep duration at baseline; model 2: model 1 plus adjustment for BMI. Abbreviations: BMI, body mass index; CI, confidence interval; HR, hazard ratio.

## Data Availability

The data are not available to be shared publicly as we do not have IRB permission for distributing the data. However, Supporting Information or data is available from the corresponding author on reasonable request.

## Supplementary Materials

The following are available online at https://www.mdpi.com/article/10.3390/jpm12010092/s1, Table S1: Hazard ratios a (95% CI) of incident hepatic steatosis or incident hepatic steatosis plus intermediate/high probability of advanced fibrosis based on FIB-4 with respect to sleep duration change and subjective sleep quality change after further adjusting for waist circumference among 75,694 participants with waist circumference available.

## Abbreviations

ALT	alanine aminotransferase
AST	aspartate aminotransferase
BMI	body mass index
CES-D	Center for Epidemiologic Studies Depression
CI	confidence interval
HOMA-IR	homeostasis model assessment of insulin resistance
HR	hazard ratio
HDL-C	high-density lipoprotein cholesterol
HPA	hypothalamic–pituitary–adrenal
hsCRP	high sensitivity C-reactive protein
LDL-C	low-density lipoprotein cholesterol
NAFLD	nonalcoholic fatty liver disease
PSQI	Pittsburgh Sleep Quality Index
