# Supplementary material for: Decrease in Sleep Duration and Poor Sleep Quality over Time Is Associated with an Increased Risk of Incident Non-Alcoholic Fatty Liver Disease
Source: J Pers Med. 2022 Jan 11;12(1):92. doi: 10.3390/jpm12010092 (PMC8777783; doi:10.3390/jpm12010092)
Supplement: Supplementary file 1 [file jpm-12-00092-s001.zip › jpm-1502478-supplementary.pdf]

Supplementary Material Table S1. Hazard ratios a (95% CI) of incident hepatic steatosis or incident hepatic steatosis plus intermediate/high probability of advanced fibrosis based on FIB-4 with respect to sleep duration change and subjective sleep quality change after further adjusting for waist circumference among 75,694 participants with waist circumference available.

	**Multivariate-adjusted HRa (95% CI) for incident hepatic steatosis**	**Multivariate-adjusted HRa (95% CI) for incident hepatic steatosis plus intermediate/high probability of advanced fibrosis based on FIB-4**
Sleep duration change category		
<-1 hour	1.21 (1.11–1.33)	1.40 (0.92–2.13)
- 1 hour	1.09 (1.04–1.15)	1.09 (0.85–1.40)
0 hour	1.00 (reference)	1.00 (reference)
1 hour	1.00 (0.95–1.06)	0.97 (0.75–1.26)
>1 hour	1.02 (0.93–1.12)	0.89 (0.53–1.49)
P for trend	< 0.001	0.142
P for quadratic term	0.005	0.731
Sleep quality change category		
Persistent good quality	1.00 (reference)	1.00 (reference)
Developed poor quality	1.01 (0.94–1.08)	1.24 (0.90–1.72)
Resolved poor quality	0.98 (0.91–1.05)	1.07 (0.75–1.52)
Persistent poor quality	1.09 (1.02–1.18)	0.94 (0.64–1.38)

[a] Estimated from Cox proportional hazards models. The multivariate model was adjusted for age, sex, center, year of screening examination, alcohol consumption, smoking, physical activity, marital status, season, history of diabetes, history of hypertension, sleep quality (only for sleep duration change category), sleep duration at baseline and waist circumference. Abbreviations: BMI, body mass index; CI, confidence interval; HR, hazard ratio.
